# Immunogenicity and safety of a live herpes zoster vaccine in hematopoietic stem cell transplant recipients

**DOI:** 10.1186/s12879-021-05806-4

**Published:** 2021-01-26

**Authors:** June Young Chun, Kichun Kim, Min Kyeong Lee, Chang Kyung Kang, Youngil Koh, Dong-Yeop Shin, Junshik Hong, Pyoeng Gyun Choe, Nam Joong Kim, Sung-Soo Yoon, Wan Beom Park, Inho Kim, Myoung-don Oh

**Affiliations:** 1 Department of Internal Medicine, Seoul National University College of Medicine, 101 Daehak-ro, Jongno-gu, Seoul, 03080 Republic of Korea; 2 Present affiliation: Department of Internal Medicine, National Cancer Center, Goyang, South Korea

**Keywords:** Herpes zoster, Varicella zoster virus, Hematopoietic stem cell transplantation, Immunogenicity, Safety

## Abstract

**Background:**

Herpes zoster (HZ) infection of hematopoietic stem cell transplant (HSCT) patients is of clinical concern. Vaccination could help restore immunity to varicella zoster virus (VZV); however, temporal changes in immunogenicity and safety of live HZ vaccines after HSCT is still unclear. The aim of this study was to elucidate the temporal immunogenicity and safety of the HZ vaccine according to time since HSCT and to determine optimal timing of vaccination.

**Methods:**

Live HZ vaccine was administered to patients 2–5 years or > 5 years post-HSCT. Control groups comprised patients with a hematologic malignancy who received cytotoxic chemotherapy and healthy volunteers. Humoral and cellular immunogenicity were measured using a glycoprotein enzyme-linked immunosorbent assay (gpELISA) and an interferon-γ (IFN-γ) enzyme-linked immunospot (ELISPOT) assay. Vaccine-related adverse events were also monitored.

**Results:**

Fifty-six patients with hematologic malignancy (41 in the HSCT group and 15 in the chemotherapy group) along with 30 healthy volunteers were enrolled. The geometric mean fold rises (GMFRs) in humoral immune responses of the 2–5 year and > 5 year HSCT groups, and the healthy volunteer group, were comparable and significantly higher than that of the chemotherapy group (3.15, 95% CI [1.96–5.07] vs 5.05, 95% CI [2.50–10.20] vs 2.97, 95% CI [2.30–3.83] vs 1.42, 95% CI [1.08–1.86]). The GMFR of cellular immune responses was highest in the HSCT 2–5 year group and lowest in the chemotherapy group. No subject suffered clinically significant adverse events or reactivation of VZV within the follow-up period.

**Conclusion:**

Our findings demonstrate that a live HZ vaccine is immunogenic and safe when administered 2 years post-HSCT.

**Supplementary Information:**

The online version contains supplementary material available at 10.1186/s12879-021-05806-4.

## Background

Herpes zoster (HZ), also called shingles (derived from Latin *cingulus* meaning girdle), is a dermatomal-vesicular disease associated with severe pain [1]. It is caused by reactivation of latent varicella zoster virus (VZV) within sensory ganglia and is more common in immunocompromised patients [1]. The incidence of HZ increases with age; the highest incidence (5–10 cases per 1000 persons) occurs in the sixth decade or beyond [2]. The burden of HZ for those with a hematopoietic stem cell transplant (HSCT) is 20–53% overall; the greatest risk (94 cases per 1000 person-years) occurs within 2 years of HSCT [3–5].

There are no clear guidelines regarding live vaccination after HSCT. With respect to HZ vaccines, limited data support vaccination after HSCT due to concerns about vaccine-induced VZV infection and lack of evidence regarding vaccine-induced immunogenicity [6–8]. Vaccination against HZ might be considered only when 24 months have elapsed since HSCT, and only in recipients showing no signs of graft-versus-host disease (GvHD) or relapse, and in those not taking immunosuppressants [9–11]. The most recent guidelines from the 2017 European Conference on Infections in Leukaemia (ECIL 7) oppose administration of live HZ vaccines; instead, they recommend antiviral agents to prevent VZV reactivation [12]. However, even with prolonged administration of antiviral agents, the incidence of HZ increases after discontinuation of prophylaxis [4, 13].

Although necessary, there is not enough evidence to support a minimum interval between transplantation and vaccination. A theoretical minimum of 24 months supposes that the HSCT recipient is immunocompetent 2 years after HSCT [14]. Here, we assessed (i) the temporal immunogenicity of live HZ vaccine in post-HSCT and (ii) the safety of live HZ vaccine in these patients.

## Methods

### Study design

This was a clinical study conducted at Seoul National University Hospital (SNUH), which is a tertiary care university-affiliated hospital in South Korea.

From July 2017 to August 2018, we prospectively enrolled patients with a hematologic malignancy who had survived with either autologous or allogeneic HSCT. Additional inclusion criteria were as follows: age older than 50 years and provision of informed consent for participation. Exclusion criteria included GvHD, use of immunosuppressants or antiviral agents, HZ reactivation within 1 year of the study period, or receipt of HZ vaccines. These patients were stratified according to time since transplantation: 2–5 years and > 5 years (hereafter referred to as HSCT 2–5 yr and HSCT > 5 yr, respectively).

Controls included patients with a hematologic malignancy who had undergone cytotoxic chemotherapy and survived without relapse for at least 6 months before enrollment (referred to as the chemotherapy group). Inclusion and exclusion criteria were applied in the same manner as for the HSCT groups. Lastly, healthy volunteers aged > 50 years without recent HZ reactivation within 1 year were recruited (referred to as the healthy group).

Study participants were given a single dose (0.65 mL) of ZOSTAVAX®. Blood samples were collected to test both humoral and cellular immune responses against VZV prior to vaccination and at 6 weeks post-vaccination. Baseline characteristics included age, sex, underlying diseases, type of HSCT or cytotoxic chemotherapy, and previous history of HZ.

### Glycoprotein ELISA (gpELISA)

Although correlates of protection (CoP) for HZ vaccines have not been defined clearly, fold rises in antibody titers in the glycoprotein enzyme-linked immunosorbent assay (gpELISA) are thought to be an excellent immune correlate of protection [15]. VZV-specific antibodies were measured quantitatively using a SERION ELISA *classic* Varicella Zoster Virus IgG kit (Institut Virion/Serion GmbH, Würzburg, Germany). This gpELISA assay uses a lentil-lectin affinity-purified preparation of glycoprotein from VZV-infected MRC-5 cells as the antigen [16, 17]. Antigen-coated 96-well plates were incubated with test sera. Human immunoglobulin G antibodies (IgGs) bound to antigen were detected by incubation with anti-human IgG antibodies. Color was developed after reaction with a substrate. Optical density was read at 405 nm.

### ELISPOT assay

Peripheral blood mononuclear cells (PBMCs) collected and frozen during the study period were tested using a BD™ IFN-γ ELISPOT kit (BD Bioscience, San Jose, CA, USA). Briefly, 96-well plates were coated with 100 μL of an anti-human-IFN-γ antibody at a concentration of 5 μg/mL overnight at 4 °C. Next, 1 × 10^6^ PBMCs/100 μL/well were activated with 5 μg/mL phytohemagglutinin (mitogen), UV-inactivated VZV culture supernatants (at a dilution of 1:80) and mock antigen supernatants for 16–20 h at 37 °C in a 5% CO_2_ humidified incubator [18]. After washing, a solution containing a biotinylated anti-human-IFN-γ detection antibody was added to each well, and streptavidin-HRP solution and substrate were used for color development. Spots were counted with a CTL-ImmunoSpot® reader (CTL ImmunoSpot, Cleveland, OH, USA) and reported as the net number of VZV-specific IFN-γ spot-forming cells (sfc) per 10^6^ PBMCs (the difference between responses to VZV antigen and control antigen) [19]. Samples lacking sufficient PMBCs and results with phytohemagglutinin responses < 300 sfc were not included in the analysis [20].

### Safety

Vaccine adverse events (AEs) were monitored based on spontaneous reports from participants, subject daily review, and history taking by investigators. All vaccinated individuals were requested to inform the study nurse or physician immediately if they noticed any serious AEs after vaccination. The participants were also requested to fill in a self-reported structured questionnaire up to 6 weeks post-vaccination and submit it on the second visit. The type and severity of local and systemic adverse events were assessed again by the study nurse 6 weeks after vaccination. Adverse events were graded on a standard scale [21]. The causality of an adverse event after immunization was classified as follows: unlikely (inconsistent), possible (indeterminate), or likely (consistent) [22].

### Statistical analysis

For continuous variables, mean (standard deviation, SD) and median (interquartile range, IQR) were used for normally and abnormally distributed data. Categorical variables were expressed as numbers and percentages. A *t*-test was used to compare continuous variables and the Chi-square or Fisher’s exact test was used to compare categorical variables. One-way ANOVA with Dunnett’s adjustment or Kruskal-Wallis test was used to calculate *P*-values for comparisons of more than two variables. All tests were two-sided and *P*-values < 0.05 were considered statistically significant. All statistical analyses were performed using SPSS for Windows (version 22; IBM Corp., Armonk, NY, USA).

## Results

### Baseline characteristics

**Fig. 1:**
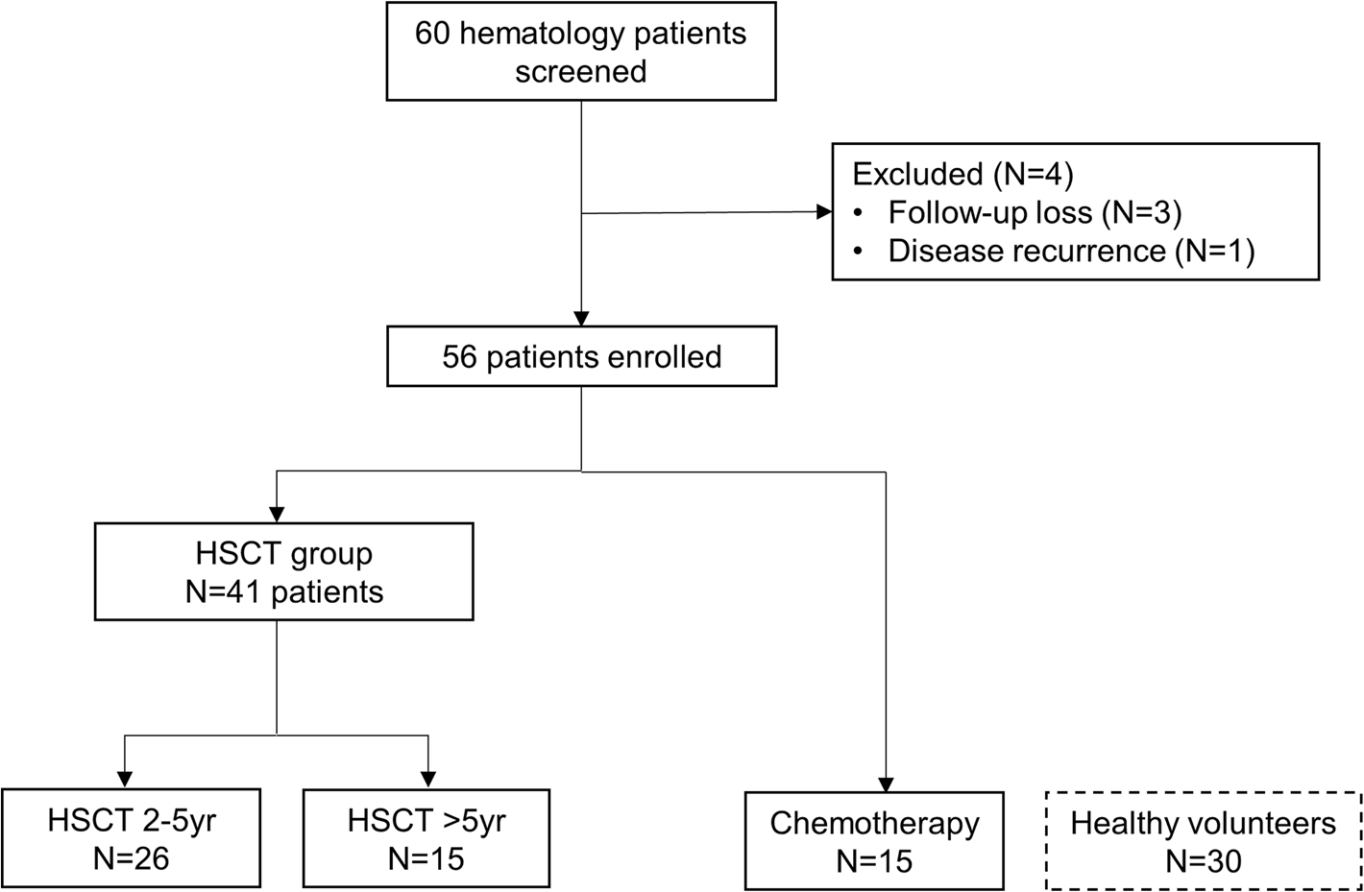
Flow diagram of study enrollment. HSCT, hematopoietic stem cell transplantation; N, number

The study screened 60 hematology patients at baseline; four patients dropped out due to follow-up loss (*n* = 3) and disease recurrence (*n* = 1). Among the remaining 56 subjects, 26 were assigned to the HSCT 2–5 yr group and 15 to the HSCT > 5 yr group. The chemotherapy group included 15 patients. In addition, 30 healthy volunteers were enrolled (Fig. 1).

Participants in each group were balanced by age; however, the male-to-female ratio of the healthy group was lower (1:2) than that in the other groups (Table 1). Leukemia and lymphoma were the most common (> 50% of participants in each group) underlying malignancies. Twenty patients in the HSCT 2–5 yr group (77%), and nine in the HSCT > 5 yr group (60%) underwent allogeneic HSCT. A history of HZ reactivation was documented in study groups. Twelve patients in the HSCT 2–5 yr group (46%), nine in the HSCT > 5 yr group (60%), and six in the chemotherapy group (40%) had shingles before enrollment in the study (Table 1). Among those reactivated, nine in the HSCT 2–5 yr group (75%), six in the HSCT > 5 yr group (67%), and five in the chemotherapy group (83%) had history within 2 years after HSCT or last cytotoxic chemotherapy.

**Table 1:** Baseline Characteristics

	HSCT 2–5 yr (*n* = 26)	HSCT > 5 yr (*n* = 15)	Chemotherapy (n = 15)^c^	Healthy (*n* = 30)
Median age, yr (IQR)	60 (55–63)	58 (53–62)	64 (57–69)	61 (57–66)
Male sex, n (%)	15 (58)	9 (60)	10 (67)	10 (33)
HSCT indication, n (%)				
Leukemia	11 (42)	4 (27)	14 (93)	N/A
Lymphoma	2 (8)	7 (47)		
Multiple myeloma	6 (23)	1 (6.5)		
Myelodysplastic syndrome	4 (15)	2 (13)		
Others^a^	3 (12)	1 (6.5)	1 (7)	
Type of HSCT, n (%)				
Allogeneic	20 (77)	9 (60)	N/A	N/A
Autologous	6 (23)	6 (40)		
Time between HSCT or last chemo and the study date, mo (IQR)	35 (25–44)	87 (66–91)	65 (23–104)	N/A
Previous shingles history, yes, n (%)	12 (46)	9 (60)	6 (40)	0 (0)
Shingles within 2 yrs after HSCT or last chemo, n (%)	9/12 (75)	6/9 (67)	5/6 (83)	N/A
Median years from shingles to the study date (IQR)	3 (1.5–4)^b^	5 (5–7)	2.5 (1.3–6)	N/A

*IQR* Interquartile range, *yr* Year, *mo* Month, *N/A* Not applicable, *chemo* Chemotherapy

^a^Aplastic anemia (n = 3), Myeloid sarcoma (n = 1), Granulocytic sarcoma (n = 1)

^b^1 patient could not remember the exact time of shingles

^c^All but one patient had acute myeloid leukemia. None had received targeted monoclonal antibody therapies

### Humoral immune responses

**Fig. 2:**
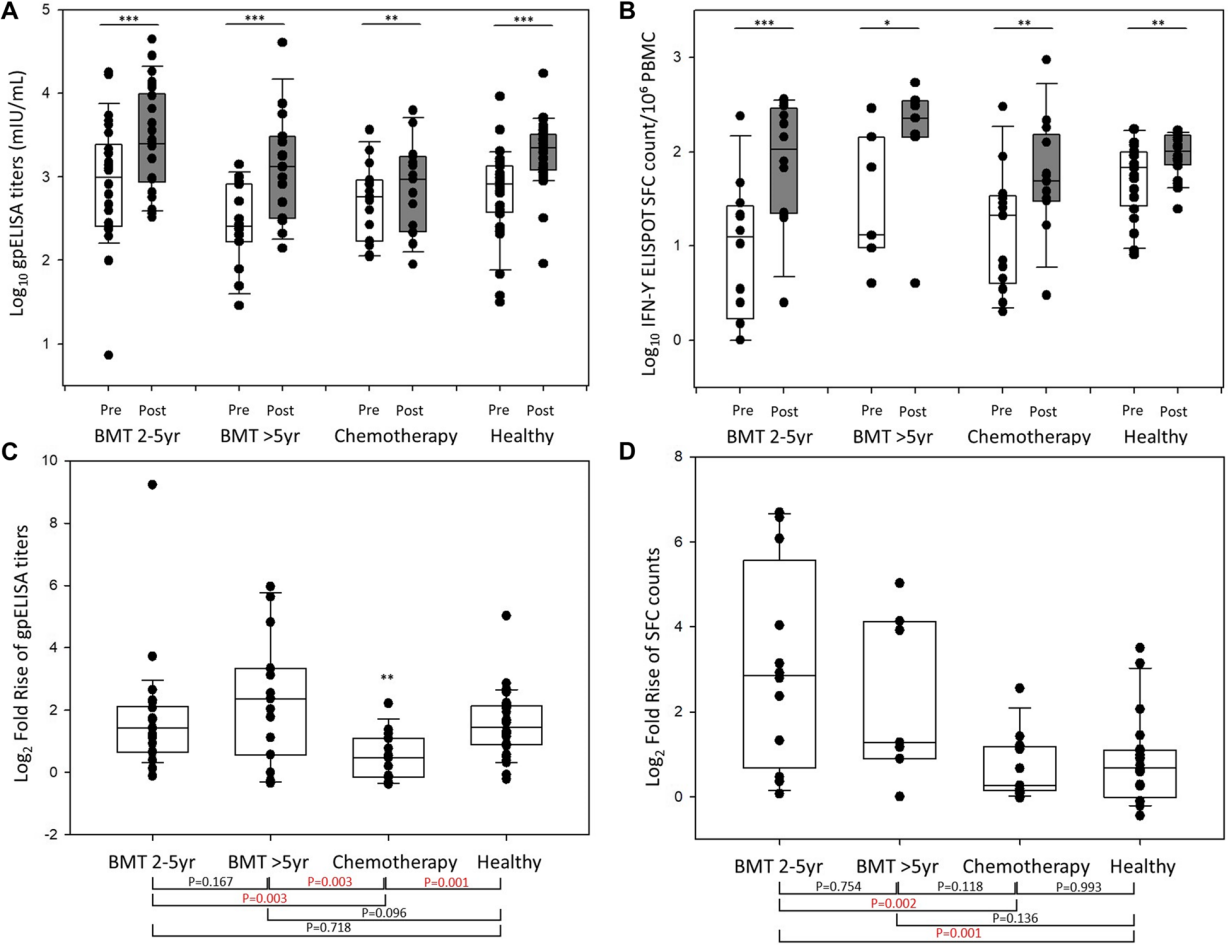
Varicella-zoster virus-specific humoral immune responses measured in a glycoprotein ELISA (gpELISA) assay before and at 6 weeks post-vaccination (**a**) and Log_2_-fold increases in the gpELISA titers (**c**) for each study group. Varicella-zoster virus-specific cellular immune responses, measured using the enzyme-linked immunospot (ELISPOT) assay, before and at 6 weeks after vaccination (**b**) and Log_2_-fold increases in spot-forming cell (sfc) counts (**d**) for each study group. The edges of the boxes represent the 25th and 75th percentiles, the horizontal lines inside the boxes are the median values, and the vertical whiskers represent the 2.5th and 97.5th percentiles (* *p* < 0.05, ** *p* < 0.01, *** *p* < 0.001)

At baseline, the gpELISA geometric mean titers (GMTs) in the HSCT 2–5 yr, chemotherapy, and healthy groups were similar (841.08, 95% CI [439.58–1609.29] vs 515.92, 95% CI [302.03–881.28] vs 657.15, 95% CI [424.18–1018.09]); values were significantly lower in the HSCT > 5 yr group (262.89, 95% CI [149.59–462.00], *p* = 0.041). At 6 weeks post-vaccination, immune responses measured in the gpELISA increased significantly in all four groups, compared with the baseline (Fig. 2a, Supplementary Table 1). The GMFR in the chemotherapy group was significantly lower than in the other groups (1.42; 95% CI, 1.08–1.86) (Fig. 2c). The GMFRs of the HSCT 2–5 yr, HSCT > 5 yr, and healthy groups were not significantly different (3.15, 95% CI [1.96–5.07] vs 5.05, 95% CI [2.50–10.20] vs 2.97, 95% CI [2.30–3.83]) (Fig. 2c). Patients with a history of shingles showed higher baseline gpELISA GMTs than those without, but the GMFRs were uninfluenced (Supplementary Table 2).

### Cellular immune responses

The ELISPOT assay was performed in only 52 subjects due to lack of qualified blood samples (HSCT 2–5 yr [*n* = 12], HSCT > 5 yr [*n* = 7], chemotherapy [*n* = 13], and healthy group [*n* = 20]). At baseline, the geometric mean concentrations (GMCs) of IFN-γ-secreting VZV-specific PBMCs were higher in the healthy group (51.39, 95% CI [34.36–76.88], *p* = 0.006) than in the other three groups (9.00, 95% CI [3.43–23.60] vs 27.99, 95% CI [8.45–92.65] vs 14.76, 95% CI [6.57–33.16]). Six weeks post-vaccination, the GMCs of IFN-γ-secreting VZV-specific PBMCs increased significantly in all groups (Fig. 2b, Supplementary Table 1). The GMFR of the IFN-γ ELISPOT assay was highest in the HSCT 2–5 yr group (8.39, 95% CI [3.30–21.32]) and lowest in the chemotherapy group (1.64, 95% CI [1.23–2.17]) (Fig. 2d). The GMFRs in the HSCT > 5 yr and healthy groups were 5.08 (95% CI, [1.86–13.86]) and 1.82 (95% CI, [1.32–2.49]), respectively. Patients with a history of shingles tended to have higher baseline GMCs than those without, but the GMFRs were uninfluenced (Supplementary Table 3).

### Safety

There were no reported cases of VZV reactivation up to 6 weeks post-vaccination in any of the study groups. Injection site reactions such as local pain, redness, edema, and itching were the most common adverse events reported by vaccine recipients (Table 2). Three patients in the HSCT 2–5 yr group (11.5%) and one each in the HSCT > 5 yr (6.6%) and chemotherapy (6.6%) groups reported vaccine-related systemic adverse events such as headache, myalgia, and fatigue (Table 2). All adverse events were mild (grade 1) and occurred within 7 days of vaccination.

**Table 2:** Adverse Events

	HSCT 2–5 yr (n = 26)	HSCT > 5 yr (n = 15)	Chemotherapy (*n* = 15)	Healthy (*n* = 30)
Any adverse events, n (%)	5 (19.2)	3 (20.0)	2 (13.3)	0 (0)
Local adverse events, n (%)	4 (15.4)	2 (13.3)	1 (6.6)	0 (0)
Pain or tenderness	3 (11.5)	2 (13.3)	1 (6.6)	0 (0)
Redness	3 (11.5)	2 (13.3)	1 (6.6)	0 (0)
Induration or edema	1 (3.8)	2 (13.3)	1 (6.6)	0 (0)
Itching	3 (11.5)	2 (13.3)	1 (6.6)	0 (0)
Systemic adverse events, n (%)	3 (11.5)	1 (6.6)	1 (6.6)	0 (0)
Headache	1 (3.8)	1 (6.6)	0 (0)	0 (0)
Myalgia or arthralgia	3 (11.5)	1 (6.6)	0 (0)	0 (0)
Fatigue	3 (11.5)	0 (0)	1 (6.6)	0 (0)
Fever	0 (0)	0 (0)	0 (0)	0 (0)
Systemic allergic reaction	0 (0)	0 (0)	0 (0)	0 (0)
Nausea or vomiting	0 (0)	0 (0)	0 (0)	0 (0)
Diarrhea	0 (0)	0 (0)	0 (0)	0 (0)

*HSCT* Hematopoietic stem cell transplantation, *N* Number

## Discussion

HZ is a high burden of disease among HSCT recipients whose immune status has been shut down and then slowly reconstituted. Here, we demonstrated that a live HZ vaccine could be administered safely to hematologic malignancy patients, and that it induced humoral and cellular immune responses as strong as those in healthy individuals. A previous study examined HZ vaccine-induced immunity in the general population, and we found that immune responses in that study were similar to those of the healthy group in this study, which raises the reliability of our study [23].

Regarding the timing of vaccination, both HSCT 2–5 yr and HSCT > 5 yr groups had comparable immune responses after HZ vaccination. Moreover, the baseline gpELISA-determined GMT was much lower in HSCT > 5 yr than any other group, highlighting the need for prompt vaccination of HSCT recipients. Interestingly, vaccine-induced humoral and cellular GMFR responses in the HSCT groups were higher than in the chemotherapy group. We also have reported that influenza vaccination induced higher immune responses in cancer patients receiving immune checkpoint inhibitors than those receiving cytotoxic chemotherapy [24, 25].

We further explored the immune responses with respect to whether or not the participant had a history of shingles. Overall, the baseline gpELISA-determined GMTs and ELISPOT-determined GMCs were higher in patients with a history of shingles than those without. ELISPOT-determined GMCs did not exhibit consistent results, which could be attributed to the small number of samples. Nonetheless, GMFRs of humoral and cellular immune responses were uninfluenced by a history of shingles.

Two previous studies of HZ vaccines administered at a median 21–27 months post-transplantation reported that it was safe, and that it reduced the incidence of VZV infection [4, 26]. Here, we found no VZV reactivation after administration of a live HZ vaccine to patients with a hematologic malignancy. Vaccine-related adverse reactions, both local and systemic, were more common in patients than in healthy volunteers; however, none were severe. Patients with hematologic malignancy might be more sensitive than healthy volunteers to adverse events and more prone to report mild symptoms. All patients recovered spontaneously within 1 week after immunization.

The study has some limitations. First, fewer patients qualified for the ELISPOT assay than for the gpELISA due to lack of sufficient blood. Inevitably, this led to less reliable results in terms of cellular immune responses. Second, patients in the HSCT > 5 yr group had fewer members that the other groups; this was due to survival and recurrence rates, chronic GvHD, and other medical issues. Third, we monitored vaccine adverse events until 6 weeks post-vaccination; a longer follow-up time is considered better for monitoring the adverse events of live vaccines, although life-threatening allergic reactions tend to appear shortly after vaccination [27].

Two inactive HZ vaccines have been introduced recently, and the published safety and efficacy data seem very strong [28, 29]. Unfortunately however, neither vaccine is available in most countries, including South Korea. Further studies of cost-effectiveness are needed. In practical terms, we expect to continue using live VZV vaccines (at least for a few years); therefore, the immunogenicity and safety data presented herein will be useful in clinical practice.

## Conclusions

In summary, the HZ vaccine could induce both humoral and cellular immune responses in patients undergoing HSCT, comparable to those in healthy volunteers. Hematologic malignancy patients who were undergoing cytotoxic chemotherapy showed weaker immune responses against the HZ vaccine. There was no reactivation of VZV during follow-up (up to 6 weeks post-immunization), and all reported adverse events were mild. These findings support current guidelines stating that live HZ vaccines may be administered to patients 2 years post-HSCT [9–11].

## Supplementary Information

**Additional file 1: Supplementary Table 1.** Humoral and cellular responses after Herpes Zoster vaccination. **Supplementary Table 2.** Humoral responses after Herpes Zoster vaccination by shingles history. **Supplementary Table 3.** Cellular responses after Herpes Zoster vaccination by shingles history.

## Data Availability

All included data are available from the corresponding author upon request.

## Abbreviations

HZ: Herpes zoster
HSCT: Hematopoietic stem cell transplant
VZV: Varicella zoster virus
GvHD: Graft-versus-host disease
gpELISA: Glycoprotein enzyme-linked immunosorbent assay
ELISPOT: Interferon-γ (IFN-γ) enzyme-linked immunospot
GMFR: Geometric mean fold rise
GMT: Geometric mean titers
GMC: Geometric mean concentrations
CI: Confidence interval
CoP: Correlates of protection
IgGs: Immunoglobulin G antibodies
PBMCs: Peripheral blood mononuclear cells
SFC: Spot-forming cells
IQR: Interquartile range
