# Supplementary material for: Immunogenicity and safety of a live herpes zoster vaccine in hematopoietic stem cell transplant recipients
Source: BMC Infect Dis. 2021 Jan 26;21:117. doi: 10.1186/s12879-021-05806-4 (PMC7836155; doi:10.1186/s12879-021-05806-4)
Supplement: Supplementary file 1 — Additional file 1: Supplementary Table 1. Humoral and cellular responses after Herpes Zoster vaccination. Supplementary Table 2. Humoral responses after Herpes Zoster vaccination by shingles history. Supplementary Table 3. Cellular responses after Herpes Zoster vaccination by shingles history. [file 12879_2021_5806_MOESM1_ESM.docx]

**Supplementary Table 1.** Humoral and cellular responses after Herpes Zoster vaccination

|  | |  | HSCT 2–5 yr  (n=26) | HSCT >5 yr  (n=15) | Chemotherapy (n=15) | Healthy  (n=30) |
| --- | --- | --- | --- | --- | --- | --- |
| gpELISA | | Sample (n) | 26 | 15 | 15 | 30 |
| GMT, mIU/mL (95% CI) | Baseline |  | 841.08  (439.58–1609.29) | 262.89  (149.59–462.00) | 515.92  (302.03–881.28) | 657.15  (424.18–1018.09) |
|  | Week 6 |  | 2653.12  (1529.78–4601.33) | 1327.03  (615.44–2861.35) | 732.57  (390.26–1375.13) | 1949.19  (1395.23–2723.11) |
| GMFR in gpELISA (95% CI) | |  | 3.15  (1.96–5.07) | 5.05  (2.50–10.20) | 1.42  (1.08–1.86) | 2.97  (2.30–3.83) |
| ELISPOT | | Sample (n) | 12 | 7 | 13 | 20 |
| GMC (95% CI) | Baseline |  | 9.00 (3.43–23.60) | 27.99 (8.45–92.65) | 14.76 (6.57–33.16) | 51.39 (34.36–76.88) |
|  | Week 6 |  | 75.51  (31.77–179.45) | 142.12  (42.12–479.50) | 54.83  (25.77–116.65) | 93.33  (73.68–118.21) |
| GMFR in ELISPOT (95% CI) | |  | 8.39  (3.30–21.32) | 5.08  (1.86–13.86) | 1.64  (1.23–2.17) | 1.82  (1.32–2.49) |

Data represent overall responses (units per mL in the gpELISA or counts per 10⁶ peripheral blood mononuclear cells in the interferon-γ ELISPOT assay) with 95% confidence intervals.

HSCT, hematopoietic stem cell transplantation; N, number; gpELISA, glycoprotein ELISA; Interferon-γ ELISPOT, interferon-γ enzyme-linked immunospot assay; GMT, geometric mean titer of varicella zoster virus-specific IgG; GMFR, geometric mean fold rise; GMC, geometric mean concentration of interferon-γ-secreting varicella zoster virus-specific peripheral blood mononuclear cells

**Supplementary Table 2.** Humoral responses after Herpes Zoster vaccination by shingles history

|  | HSCT 2–5 yr | HSCT >5 yr | Chemotherapy |
| --- | --- | --- | --- |
| Baseline GMT, mIU/mL (95% CI) | | | |
| Total | N=26 | N=15 | N=15 |
|  | 841.08 (439.58–1609.29) | 262.89 (149.59–462.00) | 515.92 (302.03–881.28) |
| Shingles history (+) | N=12 | N=9 | N=6 |
|  | 2069.90 (982.45–4361.04) | 382.60 (214.12–683.65) | 647.20 (286.85–1460.21) |
| Shingles history (–) | N=14 | N=6 | N=9 |
|  | 388.69 (166.44–907.71) | 149.74 (54.78–409.26) | 443.55 (213.76–920.39) |
|  |  |  |  |
| GMFR at Week 6 (95% CI) |  |  |  |
| Total | N=26 | N=15 | N=15 |
|  | 3.15 (1.96–5.07) | 5.05 (2.50–10.20) | 1.42 (1.08–1.86) |
| Shingles history (+) | N=12 | N=9 | N=6 |
|  | 3.06 (2.12–4.41) | 6.19 (2.75–13.92) | 1.64 (1.00–2.67) |
| Shingles history (–) | N=14 | N=6 | N=9 |
|  | 3.24 (1.40–7.49) | 3.72 (0.99–14.04) | 1.29 (0.93–1.79) |

Data represent overall responses (units per mL in the gpELISA) with 95% confidence intervals.

HSCT, hematopoietic stem cell transplantation; N, number; gpELISA, glycoprotein ELISA; GMT, geometric mean titer of varicella zoster virus-specific IgG; GMFR, geometric mean fold rise

**Supplementary Table 3.** Cellular responses after Herpes Zoster vaccination by shingles history

|  | HSCT 2–5 yr | HSCT >5 yr | Chemotherapy |
| --- | --- | --- | --- |
| Baseline GMC (95% CI) |  |  |  |
| Total | N=12 | N=7 | N=13 |
|  | 9.00 (3.43–23.60) | 27.99 (8.45–92.65) | 14.76 (6.57–33.16) |
| Shingles history (+) | N=7 | N=4 | N=5 |
|  | 11.34 (2.92–44.06) | 12.54 (3.86–40.77) | 42.44 (12.21–147.52) |
| Shingles history (–) | N=5 | N=3 | N=8 |
|  | 6.51 (1.53–27.68) | 81.62 (12.92–515.85) | 7.63 (3.41–17.07) |
|  |  |  |  |
| GMFR at Week 6 (95% CI) |  |  |  |
| Total | N=12 | N=7 | N=13 |
|  | 8.39 (3.30–21.32) | 5.08 (1.86–13.86) | 1.64 (1.23–2.17) |
| Shingles history (+) | N=7 | N=4 | N=5 |
|  | 7.61 (1.86-31.23) | 5.76 (1.18–28.21) | 1.34 (0.95–1.89) |
| Shingles history (–) | N=5 | N=3 | N=8 |
|  | 9.61 (2.79-33.12) | 4.29 (1.07–17.15) | 1.85 (1.25–2.75) |

Data represent overall responses (counts per 10⁶ peripheral blood mononuclear cells in the interferon-γ ELISPOT assay) with 95% confidence intervals.

HSCT, hematopoietic stem cell transplantation; N, number; Interferon-γ ELISPOT, interferon-γ enzyme-linked immunospot assay; GMC, geometric mean concentration of interferon-γ-secreting varicella zoster virus-specific peripheral blood mononuclear cells; GMFR, geometric mean fold rise
